# Hydrogen peroxide induces vasorelaxation by enhancing 4-aminopyridine-sensitive Kv currents through *S*-glutathionylation

**DOI:** 10.1007/s00424-014-1513-3

**Published:** 2014-04-23

**Authors:** Sang Woong Park, Hyun Ju Noh, Dong Jun Sung, Jae Gon Kim, Jeong Min Kim, Shin-Young Ryu, KyeongJin Kang, Bokyung Kim, Young Min Bae, Hana Cho

**Affiliations:** 1 Department of Physiology, Institute of Functional Genomics, Research Institute of Medical Science, School of Medicine, Konkuk University, Choongju, 380-701 Korea; 2 Division of Sport Science, College of Science and Technology, Konkuk University, Choongju, Korea; 3 Department of Physiology and Samsung Biomedical Research Institute, School of Medicine, Sungkyunkwan University, Suwon, Korea; 4 Department of Physiology and Biomembrane Plasticity Research Center, College of Medicine, Seoul National University, Seoul, Korea; 5 Department of Anatomy and Cell Biology, School of Medicine, Sungkyunkwan University, Suwon, Korea

**Keywords:** H_2_O_2_, Kv channel, Mesenteric artery, *S*-glutathionylation, Oxidative stress

## Abstract

Hydrogen peroxide (H_2_O_2_) is an endothelium-derived hyperpolarizing factor. Since opposing vasoactive effects have been reported for H_2_O_2_ depending on the vascular bed and experimental conditions, this study was performed to assess whether H_2_O_2_ acts as a vasodilator in the rat mesenteric artery and, if so, to determine the underlying mechanisms. H_2_O_2_ elicited concentration-dependent relaxation in mesenteric arteries precontracted with norepinephrine. The vasodilatory effect of H_2_O_2_ was reversed by treatment with dithiothreitol. H_2_O_2_-elicited vasodilation was significantly reduced by blocking 4-aminopyridine (4-AP)-sensitive Kv channels, but it was resistant to blockers of big-conductance Ca^2+^-activated K^+^ channels and inward rectifier K^+^ channels. A patch-clamp study in mesenteric arterial smooth muscle cells (MASMCs) showed that H_2_O_2_ increased Kv currents in a concentration-dependent manner. H_2_O_2_ speeded up Kv channel activation and shifted steady state activation to hyperpolarizing potentials. Similar channel activation was seen with oxidized glutathione (GSSG). The H_2_O_2_-mediated channel activation was prevented by glutathione reductase. Consistent with *S*-glutathionylation, streptavidin pull-down assays with biotinylated glutathione ethyl ester showed incorporation of glutathione (GSH) in the Kv channel proteins in the presence of H_2_O_2_. Interestingly, conditions of increased oxidative stress within MASMCs impaired the capacity of H_2_O_2_ to stimulate Kv channels. Not only was the H_2_O_2_ stimulatory effect much weaker, but the inhibitory effect of H_2_O_2_ was unmasked. These data suggest that H_2_O_2_ activates 4-AP-sensitive Kv channels, possibly through *S*-glutathionylation, which elicits smooth muscle relaxation in rat mesenteric arteries. Furthermore, our results support the idea that the basal redox status of MASMCs determines the response of Kv currents to H_2_O_2_.

**Electronic supplementary material:**

The online version of this article (doi:10.1007/s00424-014-1513-3) contains supplementary material, which is available to authorized users.

## Introduction

Reactive oxygen species (ROS) are detrimental to biological processes and contribute to disease conditions such as inflammation, ischemia–reperfusion injury, atherosclerosis, diabetes mellitus, and hypertension. However, some ROS like hydrogen peroxide (H_2_O_2_) may act as physiological signaling molecules and contribute to biologically beneficial processes [34, 72].

H_2_O_2_ has been suggested to be an endothelium-derived hyperpolarizing factor (EDHF) [69], a major component of endothelium-dependent relaxation in resistance-sized arteries [70]. The cellular and molecular mechanisms by which H_2_O_2_ elicits vasodilation remain to be determined, although smooth muscle hyperpolarization seems to be required [58]. The vascular smooth muscle cells functionally express four different K^+^ channels [4, 33, 43, 45, 49], namely, voltage-gated (Kv), Ca^2+^-activated (K_Ca_), ATP-sensitive (K_ATP_), and inward rectifier K^+^ (Kir) channels. The K^+^ channels are thought to play an important role in maintaining the membrane potential of vascular myocytes [45] and have been implicated in H_2_O_2_-induced smooth muscle relaxation. However, there is no general agreement on the effects of H_2_O_2_ on K^+^ channels in smooth muscle. Several studies have identified K_Ca_ channels as putative targets that are activated in the process of H_2_O_2_-induced vasodilation [81], while some other groups indicate that H_2_O_2_ induced a vasorelaxation through opening of ATP-dependent K^+^ (K_ATP_) channels [74]. The Kv channel is also suggested to be a target of H_2_O_2_, which activates Kv channels in coronary and pulmonary vascular smooth muscles [44, 54, 57]. However, Kv channels are inhibited by H_2_O_2_ in ductus arteriosus smooth muscles [2]. Studies using cloned Kv1.5, a major component of Kv current in coronary arteries, show that H_2_O_2_ increases Kv1.5 current for voltages < +20 mV but decreases it for high depolarizing voltages [12]. It is still uncertain whether H_2_O_2_ acts as a vasodilator. Studies have reported H_2_O_2_ as a vasoconstrictor [30, 67] and vasodilator [29, 73], or both [18, 38]. These differences may depend on experimental design and the specific vascular bed or vessel being studied [42, 47].

H_2_O_2_ is a small stable molecule carrying no charge, which allows it to readily cross membranes and travel freely to targets within cells [25]. Various cellular modifications occur with the increase in H_2_O_2_, and increasing evidence suggests that *S*-glutathionylation predominates in myocytes because glutathione (GSH) is the most abundant, low molecular mass, reducing equivalent [59]. H_2_O_2_ is capable of oxidizing the thiol groups of cysteine residues to form disulfide bonds with GSH (*S*-glutathionylation). *S*-glutathionylated proteins result from thiol/disulfide exchange between protein thiols and oxidized form of glutathione (GSSG) or *S*-glutathionylated protein. Modulation of protein activity by *S*-glutathionylation is a newly recognized posttranslational regulatory mechanism [20, 21]. This process results in major changes to protein conformation and function [22]. The Kir6.1/SUR2B channel and Cav1.2 channel are subject to *S*-glutathionylation induced by H_2_O_2_ [63, 71, 78].

In a previous study, we demonstrated that in mesenteric arteries, the 4-aminopyridine (4-AP)-sensitive Kv currents play a critical role in the regulation of smooth muscle resting membrane potential (Em) and vascular tone [3, 66]. In this study, we examined the hypothesis that H_2_O_2_ relaxes rat mesenteric arteries by *S*-glutathionylation-dependent activation of 4-AP-sensitive Kv channels. We performed studies using an approach of combined molecular biology, electrophysiology, and isometric organ chamber mechanics. Our results show that H_2_O_2_ enhanced the activity of 4-AP-sensitive Kv channels, possibly through *S*-glutathionylation, leading to vasorelaxation in the mesenteric artery. We also present evidence to show that Kv channels under conditions of persistent oxidative stress were not activated, but rather inhibited by the addition of H_2_O_2_, suggesting that H_2_O_2_ may act as a vasoconstrictor under certain pathological conditions.

## Methods

### Tissue and cell preparation

Male Sprague–Dawley (SD) rats (9–11 weeks old) were used for the experiments. All experiments were conducted in accordance with the National Institutes of Health guidelines for the care and use of animals, and the Institutional Animal Care and Use Committee of Konkuk University approved this study. Rats were euthanized by exposure to a rising concentration of carbon dioxide or exsanguinated by cutting the carotid arteries under deep ketamine–xylazine anesthesia. Single-cell suspensions of mesenteric arterial smooth muscle cells (MASMCs) were prepared as described previously [3]. Briefly, the second- to fourth-order branches of the superior mesenteric arteries were carefully removed and placed in normal Tyrode (NT) solution (143 mM NaCl, 5.4 mM KCl, 0.33 mM NaH_2_PO_4_, 1.8 mM CaCl_2_, 0.5 mM MgCl_2_, 5 mM hydroxyethyl piperazineethanesulfonic acid (HEPES), and 11 mM glucose, adjusted to pH 7.4 with NaOH). The arteries were cut into small pieces and transferred to a digestion solution. The tissue was first digested for 15 min in Ca^2+^-free normal NT solution containing 1 mg/mL papain (Sigma Chemical, St. Louis, MO, USA), 1 mg/mL bovine serum albumin, and 1 mg/mL dithiothreitol. The nominally Ca^2+^-free NT was prepared by omitting 1.8 mM CaCl_2_ from the NT solution. Subsequently, the tissue sample was incubated for 25 min in a second digestion solution, in which 3 mg/mL collagenase (Wako, Osaka, Japan) replaced papain. Following enzyme treatment, the cells were isolated by gentle agitation with a fire-polished glass pipette in Ca^2+^-free NT solution.

### Solutions and drugs

NT was used as the bathing solution for the patch-clamp experiments. The pipette internal solution contained 140 mM KCl, 5 mM NaCl, 5 mM MgATP, 10 mM HEPES, and 10 mM 1,2-*bis*(aminophenoxy)ethane-*N*,*N*,*N*′,*N*′-tetraacetic acid, adjusted to pH 7.2 with KOH. Bicarbonate-buffered physiological salt solution (PSS) was used as the bath solution for the organ chamber mechanics experiments. The PSS was composed of 136.9 mM NaCl, 5.4 mM KCl, 1.5 mM CaCl_2_, 1.0 mM MgCl_2_, 23.8 mM NaHCO_3_, and 0.01 mM EDTA. All chemicals, including H_2_O_2_, GSH, and GSSG, were purchased from Sigma.

### Electrophysiological recordings

We used the conventional whole-cell configuration of the patch-clamp technique [3] to record membrane currents and Em. EPC8 (HEKA, Mahone Bay, Nova Scotia, Canada) patch-clamp amplifier with a DAQPad-6070E interface (National Instrument, Austin, TX, USA) or an Axopatch 200B patch-clamp amplifier with a DigiData 1200 interface (Axon Instruments, Foster City, CA, USA) was used. Data were digitized with custom-built software (R-clamp, by Dr. SY Ryu) or with pClamp6 software (Axon Instruments) at a sampling rate of 1–10 kHz. The data were low-pass filtered at 1 kHz and saved for analysis. Voltage pulse generation was also controlled by R-clamp software and pClamp6. Patch pipettes were pulled from borosilicate capillary tubes (Clark Electromedical Instruments, Pangbourne, UK) using a puller (PP-83; Narishige, Tokyo, Japan). We used patch pipettes with a resistance of 2–4 MΩ when filled with the abovementioned pipette solution. Recordings were started at least 7 min after establishing the whole-cell configuration to allow adequate cell dialysis of the pipette solution. All experiments were carried out at room temperature (20–25 °C).

Kv currents were elicited by depolarizing steps between −60 and +50 mV (200 ms duration) from a holding potential of −70 mV. Tetraethylammonium (TEA, 1 mM) was added to all bath solutions during the patch-clamp study to prevent activation of big-conductance K_Ca_ (BK_Ca_) channels. Additionally, specific activation of the Kv current was confirmed using 4-AP at the end of the experiments. Conductance–voltage (*G*–*V*) relationships were plotted using steady state current amplitudes divided by driving force (Em–Erev, where Erev is the reversal potential of the Kv current). The normalized conductance was fit using Origin 6.0 software to the Boltzmann equation.

### Organ chamber isometric contraction measurements

The mesenteric arterial rings were mounted vertically on two L-shaped stainless steel wires in a 3-mL tissue chamber for the tension measurements. One wire was attached to a micromanipulator and the other to an isometric force transducer (FT03; Grass, West Warwick, RI, USA). Changes in isometric force were digitally acquired at 1 Hz with a PowerLab data acquisition system (AD Instruments, Colorado Springs, CO, USA). Resting tension was set to 1 g using the micromanipulator. After a 60-min equilibration under resting tension in a tissue chamber filled with PSS, the rings were sequentially exposed to 70 mM KCl–PSS (10 min) and PSS (15 min) three times for stabilization. The high KCl (70 mM)–PSS was prepared by replacing NaCl with equimolar KCl in PSS. Bath solutions were thermostatically controlled at 37 °C and were continuously saturated with a mixture of 95 % O_2_ in 5 % CO_2_ to achieve pH 7.4.

### Western blot

Primary cultures of rat MASMCs between six and ten passages were used for Western blot. MASMCs were isolated from SD rats and cultured in Dulbecco’s modified Eagle’s medium (DMEM) containing 10 % fetal bovine serum (FBS) and 1 % penicillin–streptomycin. The cells were grown to 80 % confluence and starved in DMEM without FBS for 12 to 24 h prior to experiments. After starvation, cells were treated with H_2_O_2_ for 10 min at 37 °C. The cells were then washed twice with phosphate-buffered saline (PBS) and lysed using RIPA buffer (TNT Research, Seoul, South Korea). Samples were run on an 8 % SDS–polyacrylamide nonreducing gel and then transferred to a polyvinylidene fluoride (PVDF) membrane (Millipore, Bedford, MA, USA). Rabbit primary antibodies against Kv1.2, Kv1.5, and Kv 2.1 (1:500; Alomone Lab) and secondary antibodies conjugated with horseradish peroxidase were used in the Western blot (1:2,000; Cell Signaling Technology, Danvers, MA, USA). Signals were visualized using Las-4000 (Fuji Film, Tokyo, Japan).

### Streptavidin pull-down assay

The culture medium was replaced with fresh medium 2 h before experiments. Biotinylated glutathione ethyl ester (BioGEE; 100 μM; Invitrogen, Carlsbad, CA, USA) was added to the medium and incubated for 1 h, followed by H_2_O_2_ (0.1 or 10 mM) challenges for 10 min. Biotin–GSH-conjugated proteins were pulled down using Dynabeads streptavidin according to the methods provided by Invitrogen. Dynabeads streptavidin was washed thrice with PBS before conjugation with biotin. Samples were then mixed with beads and incubated at room temperature with gentle rotation for 30 min. A magnet was used to separate the biotinylated molecule–bead complex. The supernatant containing unlabeled proteins was discarded, and the pellet was resuspended, followed by washes with PBS. The biotinylated molecule–bead complex was boiled with loading buffer for 7 min for Western blotting.

### Data analysis

The Origin 6.0 software (Microcal Software, Inc., Northampton, MA, USA) was used for data analysis. Activation kinetics was calculated by fitting the data to a single exponential decay function. The time course of current inactivation was also fit to a single exponential function. The results are shown as mean ± standard error. Paired or independent Student’s *t* tests were used to test for significance, and *p* < 0.05 was regarded as significant. We performed one-way repeated measures ANOVA and Holm–Sidak test in order to examine the statistical significance of data shown in Fig. 3b and one-way ANOVA and Tukey’s test for Fig. 5c using SigmaPlot 12.5.

## Results

### H_2_O_2_ causes relaxation of the precontracted mesenteric arterial rings by redox-dependent alterations

**Fig. 1:**
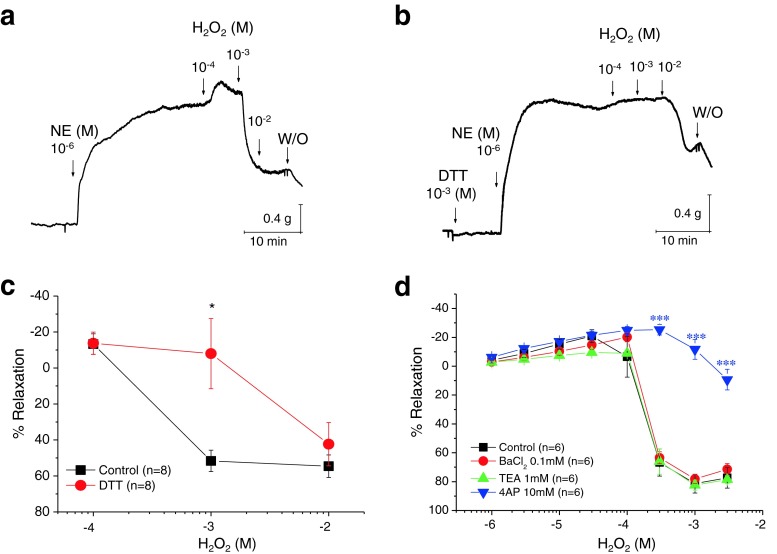
Effects of H_2_O_2_ on NE-precontracted mesenteric arterial rings under control and DTT-pretreated conditions. **a** Isometric tension recordings showing the effects of increasing concentrations of H_2_O_2_ (0.1–10 mM) on mesenteric arteries precontracted with NE. **b** Pretreatment with DTT inhibited the H_2_O_2_-induced relaxation. **c** H_2_O_2_-induced relaxation under control and DTT-pretreated conditions. The H_2_O_2_ effect was blocked by DTT (*p < 0.05 vs. control condition). **d** Summary of the effects of various potassium current blockers on the H_2_O_2_-induced relaxation (***p < 0.001 vs. control)

We used isometric organ chamber mechanics to examine whether H_2_O_2_ relaxes rat mesenteric arteries. We used arterial rings without intact endothelium. The arterial rings were precontracted with norepinephrine (NE) (1 μM). H_2_O_2_ induced a concentration-dependent relaxation in the precontracted mesenteric arterial rings (Fig. 1a, c, d). We determined whether a thiol-specific reducing agent, DL-dithiothreitol (DTT), could reverse H_2_O_2_-induced relaxation. Pretreatment with 1 mM DTT almost completely prevented the relaxation by 1 mM H_2_O_2_ (Fig. 1b, c). The addition of DTT to vascular rings, in the absence of contractile agonist, did not affect resting tension (0.23 ± 0.19 vs. 0.22 ± 0.19 g before and after adding 1 mM DTT, respectively; *n* = 8). These data indicate that the effect of DTT is specific for H_2_O_2_-induced relaxation and suggest that thiol groups in smooth muscle are targets of H_2_O_2_ signaling. TEA was used to assess the contribution of the K^+^ channels to H_2_O_2_-induced smooth muscle relaxation. At a concentration of 1 mM, TEA is reported to be relatively specific for BK_Ca_ channels and has little effect on voltage-dependent K^+^ channels [65]. One millimolar TEA (Fig. 1d; *n* = 6) did not significantly inhibit the relaxation caused by H_2_O_2_ (compared with control; *n* = 6). BaCl_2_ (100 μM), a blocker of Kir, did not affect the H_2_O_2_-induced relaxation either (Fig. 1d). We attempted similar experiments with 10 mM 4-AP, a known Kv channel blocker; 4-AP significantly attenuated H_2_O_2_-induced relaxation (Fig. 1d, *n* = 6). These data suggest that 4-AP-sensitive Kv channels mediate the vasodilation by H_2_O_2_ in the mesenteric artery.

### Effects of H_2_O_2_ on Kv currents in rat MASMCs

**Fig. 2:**
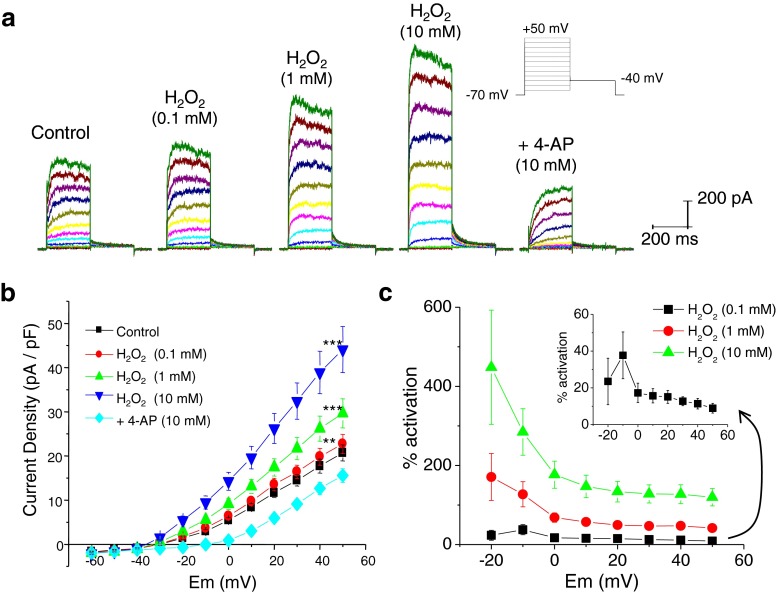
H_2_O_2_ increases Kv currents in MASMCs in a concentration-dependent manner. **a** Representative current traces illustrating the effect of H_2_O_2_ (0.1–10 mM) on whole-cell Kv currents. Deactivating tail currents were observed at −40 mV, following various test potentials, indicative of Kv channel activation. Application of 4-AP (10 mM) reduced current below the baseline level. **b** Current–voltage (*I*–*V*) relationship with H_2_O_2_ treatment (0.1–10 mM) (*n* = 19 MASMCs; **p < 0.01 vs. control; ***p < 0.001 vs. control). **c** H_2_O_2_-induced activation of Kv current. Amplitudes of Kv currents were measured at +40 mV. The magnitude of activation with various concentrations of H_2_O_2_ was plotted against Em (*n* = 19 for each concentration). *Insets* show the indicated graph with expanded scale

To examine whether H_2_O_2_ activated 4-AP-sensitive Kv currents, we recorded Kv currents using the conventional whole-cell, patch-clamp technique with depolarizing voltage steps as described previously [3] (Fig. 2). Cells were held at −70 mV to remove voltage-dependent channel inhibition, and membrane potential was stepped from −60 to +50 mV in 10-mV increments. Cumulative application of H_2_O_2_ superfusion (5 min at each concentration) increased Kv currents (Fig. 2a). Addition of 10 mM 4-AP to the bath reduced current magnitude below the baseline level, indicating that 4-AP-sensitive Kv channels are responsible for the outward current. H_2_O_2_-induced Kv current modulation was concentration-dependent; 0.1, 1, and 10 mM H_2_O_2_ induced an increase of Kv current amplitude at +40 mV to 11.4 ± 2.9, 47.5 ± 7.7, and 127.4 ± 23.8 %, respectively (Fig. 2b, c). Analysis of *I*–*V* relationship also indicated that the effect of H_2_O_2_ on current becomes significant at −20 mV. The degree of activation of the steady state Kv currents by H_2_O_2_ was large in the negative voltage range compared to those at potentials positive to 0 mV (Fig. 2c). This indicates that H_2_O_2_ can act as a potent modulator of the Kv channel function in rat MASMCs within the range of physiologically relevant voltages. H_2_O_2_, even at 10 mM, did not result in any nonspecific effects due to cellular damage. In all cells tested, neither access resistance nor leak current was significantly altered (Fig. 2a, b).

**Fig. 3:**
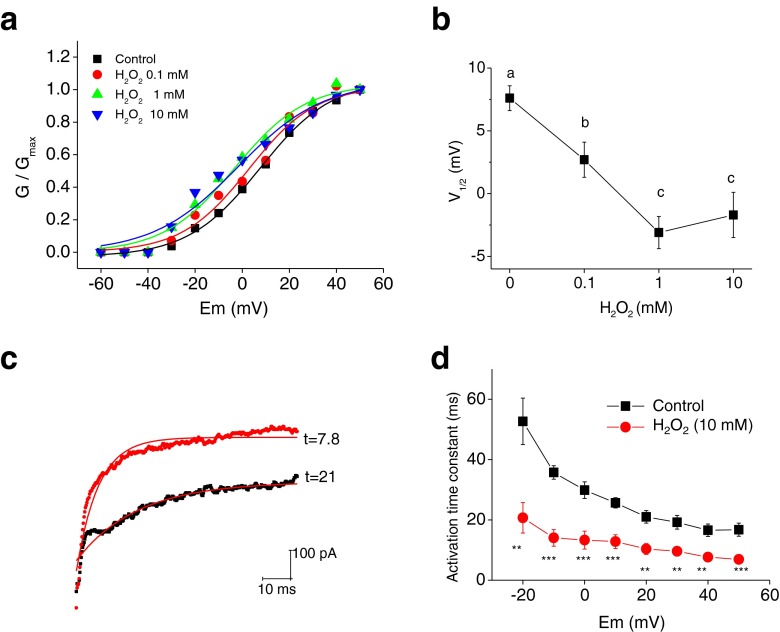
H_2_O_2_ shifts *G*–*V* curves for Kv channels to the left and speeds up channel activation. **a** *G*–*V* curves for K_V_ channels, before and after H_2_O_2_ (0.1–10 mM) treatment. Smooth curves were fitted using the Boltzmann function. **b** *V*_1/2_ obtained from the Boltzmann function is plotted against H_2_O_2_ concentration. One-way repeated measures ANOVA and Holm–Sidak test (*α* = 0.01, *p* < 0.05). Statistically distinct groups are indicated by letters. **c** Representative traces at +40 mV show that H_2_O_2_ (10 mM) accelerates Kv current activation. Activation of K_V_ currents was fit with a single exponential function. Fits to original traces and time constant (τ) values are shown. **d** Time constants are presented as a function of the pulse potential in control (*filled black square*) and following application of 10 mM H_2_O_2_ (*filled red circle*) (*p < 0.05; **p < 0.01; ***p < 0.001 vs. control)

We then examined whether H_2_O_2_ influences voltage dependence of activation and activation kinetics of the putative Kv current (Fig. 3). We noticed that the conductance–voltage (*G*–*V*) curves of the Kv channel were significantly shifted to more negative potentials after H_2_O_2_ application (Fig. 3a), i.e., the channel was now activated at a more hyperpolarized potential. *V*_1/2_ (midpoint of the *G*–*V* curve) in control and 0.1, 1, and 10 mM H_2_O_2_ were 7.6 ± 1.0, 2.7 ± 1.4, −3.1 ± 1.3, and −1.7 ± 1.8 mV, respectively (*n* = 14; Fig. 3b). The slope factors were unaffected (Fig. 3a). H_2_O_2_ also had an important effect on the time course of activation of the Kv channel (Fig. 3c). For example, for a −20-mV depolarizing pulse, the time constant of activation decreased by 60 %. Similar effects were observed for all depolarizing pulses tested (*n* = 16, Fig. 3d), suggesting that H_2_O_2_ speeds up the activation of Kv currents. The data from Figs. 2 and 3 (i.e., 4-AP sensitivity, voltage dependence, and kinetics) suggest that the currents modulated by H_2_O_2_ are 4-AP-sensitive K_V_ currents.

### Biochemical evidence for the Kv channel *S*-glutathionylation by H_2_O_2_

**Fig. 4:**
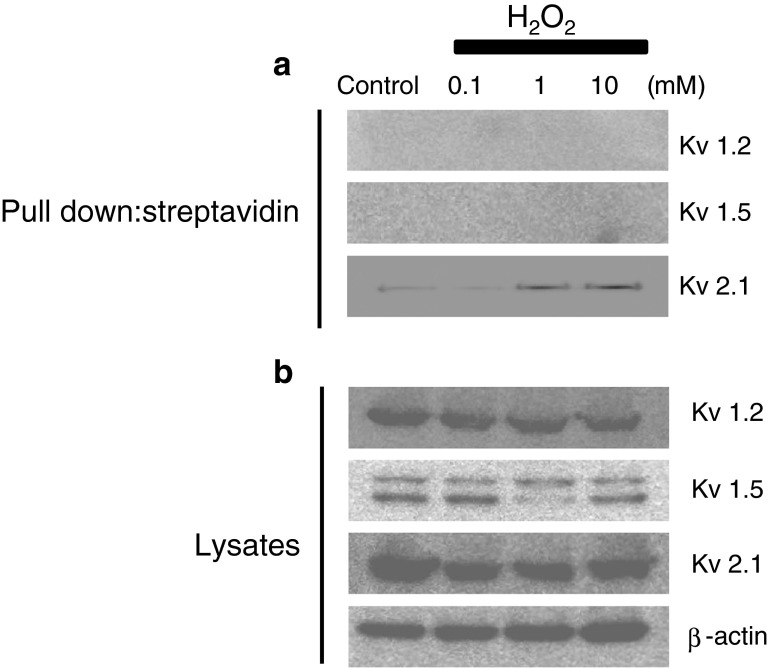
*S*-glutathionylation of the Kv 2.1 channel after exposure to H_2_O_2_. **a** Kv 2.1 channels were detected only from samples that were obtained from the cells treated with both BioGEE and H_2_O_2_ in the streptavidin pull-down assay. In the streptavidin pull-down assay, Kv1.2 and Kv1.5 channels were not detected even from samples that were obtained from cells treated with both BioGEE and H_2_O_2_. **b** Kv channel subunits were detected through conventional Western blot using rat mesenteric arterial smooth muscle cell (MASMC) primary cultures pretreated with various concentrations of H_2_O_2_ and untreated control. Band density did not change with H_2_O_2_ treatment. The results are representative examples of three independent experiments

To reveal the mechanism(s) by which H_2_O_2_ increases the voltage sensitivity and activation kinetics of 4-AP-sensitive Kv channels, we examined whether the Kv channel proteins from rat MASMCs can be glutathionylated after exposure to H_2_O_2_. It has been reported that Kv1.2 and Kv1.5 channels are molecular identities of 4-AP-sensitive Kv channels in vascular smooth muscle cells. In addition, a recent study reported that Kv2.1 and Kv 9.3 channels are oxygen-sensitive K^+^ channels in the pulmonary artery. Since a previous study confirmed the presence of Kv1.2, Kv1.5, and Kv2.1 channel proteins in isolated MASMCs by immunocytochemistry [75], we examined the *S*-glutathionylation of these channel proteins by using a streptavidin pull-down assay. Primarily cultured rat MASMCs were loaded with BioGEE (250 μM) for 1 h, followed by H_2_O_2_ (0.1–10 mM) challenge for 10 min, as described previously [82]. Strong Kv1.2 (75 kDa), Kv1.5 (67 kDa), and Kv2.1 (95 kDa) bands were detected in the whole-cell lysates (Fig. 4, lower panel). If BioGEE was incorporated into channel proteins, streptavidin beads should pull down the channel protein–BioGEE complex, which would be further detected by channel protein antibodies in a Western blot. In contrast, if the channel proteins were not glutathionylated, the binding of the channel protein to BioGEE should decrease, resulting in a weaker band or even no band in the Western blot. In the streptavidin pull-down experiments, the immunoreactivity of Kv2.1 was significantly increased in the cell lysate pretreated with H_2_O_2_, compared to control cells (Fig. 4, upper panel). After streptavidin pull-down, immunoreactivities of Kv1.2 and Kv1.5 were not detectable. Western blotting of whole lysates verified Kv1.2 and Kv1.5 protein expression. Similar results were obtained using immunoprecipitation with anti-GSH, followed by immunoblot with antibodies against Kv1.2, Kv1.5, and Kv2.1 (data not shown).

### *S*-glutathionylation mediates activation of Kv currents by H_2_O_2_

**Fig. 5:**
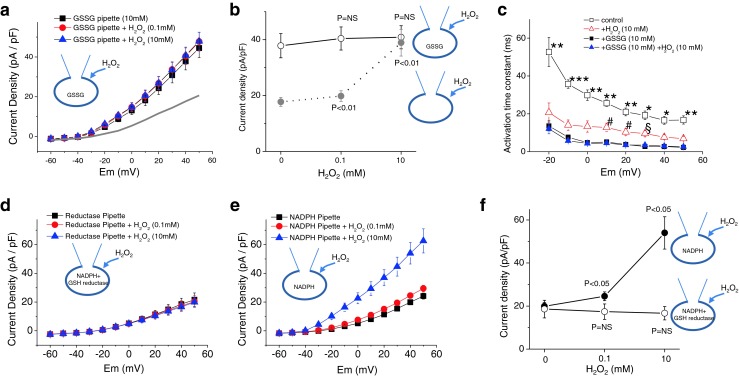
GSSG increases Kv currents in rat MASMCs. **a** Effects of adding GSSG in the pipette solution on Kv currents and on the action of H_2_O_2_. Averaged *I*–*V* curves before (*filled black square*) and after bath application of 0.1 mM H_2_O_2_ (*filled red circle*) or 10 mM H_2_O_2_ (*filled blue triangle*) in cells with GSSG in the pipette are shown. *Gray line* indicates the averaged *I*–*V* relationship with control pipette solution (redrawn from Fig. 2b for comparison). H_2_O_2_ was added cumulatively (*n* = 12). Note that Kv currents with pipette solution containing GSSG (*filled black square*) are much larger than those with control pipette solution (*gray line*). **b** Comparative data of Kv current densities measured at +40 mV for H_2_O_2_ effects in control pipette solution (*filled gray circle*; *n* = 21) with that in the pipette containing 10 mM GSSG (*empty circle*; *n* = 12). *p* < 0.01 vs. basal condition (control pipette solution); *p* = NS vs. basal condition (GSSG in the pipette). **c** Time constants recorded with GSSG pipette are presented as a function of the pulse potential before (*filled black square*) and after application of 10 mM H_2_O_2_ (*filled blue triangle*) (*n* = 8). Time constants recorded with control pipette (*empty square* and *empty triangle*) are redrawn from Fig. 3d for comparison (*n* = 16). One-way ANOVA and Tukey’s test, *p < 0.05; **p < 0.01; ***p < 0.001 vs. all other groups; ^#^ *p* < 0.05 vs. GSSG pipette groups; ^§^ *p* < 0.05 vs. GSSG alone. **d**, **e** Effects of GSH reductase (0.2 units/mL) plus NADPH (**d**) on Kv currents and on the action of H_2_O_2_. NADPH alone had no effect (**e**). Averaged *I*–*V* curves before (*filled black square*) and after bath application of 0.1 mM H_2_O_2_ (*filled red circle*) and 10 mM H_2_O_2_ (*filled blue triangle*) are shown. **f** Comparative data between Kv current densities measured at +40 mV for H_2_O_2_ effects in the pipette containing NADPH alone (*filled black circle*; *n* = 11) and those in the pipette containing NADPH plus GSH reductase (*empty circle*; *n* = 11). *p* = NS vs. basal condition (NADPH plus GSH reductase)

GSSG causes *S*-glutathionylation [20]. We examined whether GSSG directly increased Kv currents. We established the conventional whole-cell configuration to deliver GSSG to the cytosol, as done in a previous study [68]. Current recordings usually started 10 min after the whole-cell configuration was made. Intracellular loading of 10 mM GSSG via a patch pipette significantly increased the Kv current (Fig. 5a). At +40 mV, Kv current densities in the absence and presence of GSSG were 17.7 ± 1.5 pA/pF (*n* = 19) and 37.8 ± 4.3 pA/pF (*n* = 21, *p* < 0.01), respectively (Fig. 5b). Furthermore, with GSSG in the pipette, subsequent application of H_2_O_2_ had no effect on Kv current (Fig. 5a, b). We then tested whether GSSG would also induce a negative shift in the activation curves for Kv channels. We found that *G*–*V* curves of the Kv channel were significantly shifted to more negative potentials after GSSG application. *V*_1/2_ in the presence of GSSG was −1.9 ± 2.2 mV (*n* = 12), and it was not significantly different from that induced by 10 mM H_2_O_2_ (−1.7 ± 1.8 mV, *n* = 14). GSSG also had an effect on the time course of activation (*n* = 8, Fig. 5c). In the presence of GSSG, the time constant of activation decreased, and the change was even larger than that observed with 10 mM H_2_O_2_. More importantly, when 10 mM H_2_O_2_ was added to the GSSG-treated cells, we observed no further change in the *G*–*V* curve (*V*_1/2_; −1.0 ± 1.6 mV, *n* = 12) and activation kinetics (Fig. 5c). These data indicate that GSSG mimicked and occluded the effects of H_2_O_2_, implying that *S*-glutathionylation appears to occur in the Kv channel during H_2_O_2_ application leading to the activation of the channel activity.

To confirm that *S*-glutathionylation mediates the activation of Kv currents by H_2_O_2_, we examined the effect of glutathione reductase on the action of H_2_O_2_. Glutathione reductase reduces GSSG to GSH and prevents oxidation of GSH. Since NADPH is an indispensable cofactor for glutathione reductase activity, NADPH (1 mM) was applied to cells together with glutathione reductase. NADPH alone did not block the stimulatory effect of H_2_O_2_ on Kv currents (Fig. 5e). However, intracellular glutathione reductase completely abolished the stimulatory effect of H_2_O_2_ on Kv currents (Fig. 5d). Data summarized in Fig. 5f suggest that increased channel activity by H_2_O_2_ occurs because of direct modification of thiol groups on the Kv channel by GSSG in rat MASMCs.

### Redox status determines the response of Kv current to H_2_O_2_

**Fig. 6:**
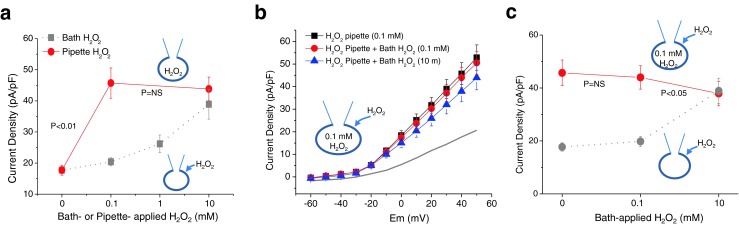
Effects of intracellular redox states on the capacity of H_2_O_2_ to stimulate Kv channels. **a** Intracellular application of H_2_O_2_ induced a concentration-dependent increase of Kv currents (*filled red circle*, *n* = 21). Compared to bath application (*filled gray square*, *n* = 21), the concentration–response relationship was shifted to the left when H_2_O_2_ was added intracellularly. Current densities of Kv channels were measured at +40 mV. **b** Effects of 100 μM H_2_O_2_ in the pipette solution on the action of H_2_O_2_. Averaged *I*–*V* curves before (*filled black square*) and after bath application of 0.1 mM H_2_O_2_ (*filled red circle*) and 10 mM H_2_O_2_ (*filled blue triangle*) are shown. *Gray line* indicates the averaged *I*–*V* relationship with control pipette solution (redrawn from Fig. 2a for comparison). Note that Kv currents with pipette solution containing 100 μM H_2_O_2_ (*filled black square*) are much larger than those with control pipette solution (*gray line*). **c** Comparative data between Kv current densities measured at +40 mV for H_2_O_2_ effects in control pipette solution (*filled gray circle*; *n* = 21) and those in the pipette containing 100 μM H_2_O_2_ (*filled red circle*; *n* = 21)

Recent studies showed that an increase of ROS is linked to hypertension [17]. Most endogenously produced ROS, including H_2_O_2_, are derived from mitochondrial respiration [23, 36], wherein 1–2 % of consumed oxygen is converted to superoxide radical and then to H_2_O_2_ [10, 15]. Since H_2_O_2_ treatment does not induce relaxation but contraction of vascular smooth muscle cells in hypertensive vessels [28], we hypothesized that the increased basal H_2_O_2_ preoccupied the activation mechanism of Kv channels, thus rendering H_2_O_2_ treatment ineffective in activating Kv channels. To mimic the endogenously generated H_2_O_2_, we directly conveyed H_2_O_2_ into the cytosol by adding H_2_O_2_ in the patch pipette. The elevated intracellular level of H_2_O_2_ increased the Kv currents (Fig. 6a). At +40 mV, 0.1 and 10 mM H_2_O_2_ increased Kv current density up to 45.7 ± 4.8 pA/pF (*n* = 21, *p* < 0.01 vs. control pipette) and 43.8 ± 3.7 pA/pF (*n* = 21, *p* < 0.01 vs. control pipette), respectively (Fig. 6a). Notably, the concentration–response relationship was shifted to the left, implying that intracellular H_2_O_2_ is more effective in elevating Kv currents than extracellular H_2_O_2_. Under this high level of intracellular H_2_O_2_, the stimulatory effect of bath-applied H_2_O_2_ was completely abolished (Fig. 6b). Summarized data in Fig. 6c showed that the basal Kv current density under 0.1 mM of intracellular H_2_O_2_ was 45.7 ± 4.8 pA/pF (*n* = 21), and it was not enhanced but unaffected (43.9 ± 4.4 pA/pF, *p* > 0.05) or rather reduced (37.9 ± 4.6 pA/pF, *p* < 0.01) by subsequent bath application of 0.1 and 10 mM H_2_O_2_, respectively. These data suggest that high levels of basal H_2_O_2_ upregulates Kv currents through *S*-glutathionylation, which may keep acute exposure to H_2_O_2_ from regulating the Kv channels.

## Discussion

It is still uncertain whether H_2_O_2_ acts as a vasodilator [55, 80]. There is even less certainty regarding the signal transduction and/or effector mechanism(s) that may be involved in the vascular response to H_2_O_2_. For example, it may involve the BK_Ca_ channel [81], K_ATP_ channel [40], or Kv channel [55]. The present study resolves some of this complexity by providing direct evidence for an effector molecule that can mediate H_2_O_2_-induced vasodilation through the Kv channel and proposes that *S*-glutathionylation underlies the stimulatory effect of H_2_O_2_ on Kv channels. Further experiments demonstrate that under oxidative conditions, Kv channels were not stimulated but rather inhibited by acute exposure to H_2_O_2_, suggesting that cellular redox status affected *S*-glutathionylation of the Kv channel and determined the response of the Kv channel to H_2_O_2_.

In the present study, H_2_O_2_ relaxed the rat mesenteric artery that was precontracted with an agonist. In contrast, H_2_O_2_ failed to relax arteries pretreated with DTT and 4-AP; 1 mM TEA and 100 μM BaCl_2_ did not affect the H_2_O_2_ response. The inability of H_2_O_2_ to relax arteries pretreated with 4-AP suggested that the relaxation response to H_2_O_2_ involved stimulation of the Kv channel, a mechanism that has also been suggested from patch-clamp/whole-cell studies employing other cell types [12, 54]. Here, we directly measured the effects of H_2_O_2_ on Kv channels in MASMCs. H_2_O_2_ increased 4-AP-sensitive Kv currents in a concentration-dependent manner. This was a result of change in the voltage dependence of activation; 10 mM H_2_O_2_ shifted voltage dependence of 4-AP-sensitive Kv channel conductance by ~7.6 mV (Fig. 3a, b), and the shift was concentration-dependent. The activation kinetics was also accelerated after exposure to H_2_O_2_ (Fig. 3c, d). It is worthy to note that unlike the role of BK_Ca_ channel in H_2_O_2_-induced dilation of coronary arteries and arterioles [5, 81], the BK_Ca_ channel did not contribute to the dilatory effect of H_2_O_2_ in rat mesenteric arteries (Fig. 1). This discrepancy may suggest that the regulatory mechanisms underlying vascular tone and the sensitivity of diverse K^+^ channels to H_2_O_2_ differ among different types of arteries. The 4-AP-sensitive Kv channels are expressed in high density in myocytes derived from rat mesenteric [75] and human pulmonary [26] arteries and are important targets of receptor agonists [3]. BK_Ca_ channels are the key determinant of coronary arterial tone [37].

Previous studies have proposed a variety of molecular pathways that can be stimulated by H_2_O_2_. For example, guanylyl cyclase may underlie H_2_O_2_ relaxation of pulmonary arteries [11], while arachidonic acid (AA) may mediate a vasodilator effect of H_2_O_2_ in coronary arteries [5]. Recent studies have shown that H_2_O_2_ induces *S*-glutathionylation of the channel protein, thereby affecting channel activity [71, 77, 78]. Since the thiol-specific reducing agent DTT blocked the vasodilatory effect of H_2_O_2_ (Fig. 1), we considered it possible that *S*-glutathionylation of the Kv channel protein mediates the stimulatory effect of H_2_O_2_ on Kv currents in the mesenteric artery smooth muscle. We found that addition of H_2_O_2_ to MASMCs increased *S*-glutathionylation of the Kv2.1 channel protein dramatically (Fig. 4). It is well known that Kv2.1 expresses a slow-inactivating, TEA-resistant, and 4-AP-sensitive Kv current in rat and human mesenteric arteries [51]. However, we could not exclude a possible glutathionylation of Kv1.2 and Kv1.5 channel proteins since it might not have been detected due to differences in pull-down efficiency in each Kv channel after *S*-glutathionylation. To confirm the cause–effect relationship, we blocked *S*-glutathionylation by using GSH reductase. GSH reductase completely abolished the stimulatory effect of H_2_O_2_. Addition of exogenous GSSG directly stimulated channel activity. Similar to H_2_O_2_, GSSG alters channel function by speeding up the activation kinetics and shifting the voltage dependence of channel activation to the left. Bath application of H_2_O_2_ (10 mM), subsequent to maximal GSSG effect, induced no further change in the Kv channel. These results suggest that *S*-glutathionylation of the Kv channel protein mediates the stimulatory effect of H_2_O_2_ on the Kv channel and, consequently, the vasodilatory effects in the mesenteric artery. Interestingly, conditions of increased oxidative stress within smooth muscle cells impaired the capacity of exogenous H_2_O_2_ to stimulate Kv channels (Fig. 6). Not only was the H_2_O_2_ stimulatory effect much weaker, but also the inhibitory effect of H_2_O_2_ was unmasked. The molecular mechanism of how H_2_O_2_ inhibits Kv channel under oxidative condition is not yet known. However, it can be speculated that since *S*-glutathionylation of the Kv channel persists and the Kv channels are already maximally enhanced, signals such as cyclooxygenase are involved [5]. Taken together, *S*-glutathionylation of the Kv channel under elevated basal H_2_O_2_ levels may be involved in the development of the pathology of the hypertensive vessel. This concept is still speculative; therefore, further studies will be required to test this hypothesis.

In the present study, a high concentration of extracellular H_2_O_2_ is required to regulate K_V_ channels. This argument holds for neurons. This can be reflected from the fact that, in the hippocampus, the IC_50_ value for extracellular H_2_O_2_ to affect postsynaptic potentials was nearly 6 mM [46]. In contrast to extracellular application, a low level of intracellular H_2_O_2_ is sufficient to elevate the Kv currents (Fig. 6). This difference suggests that either the permeability of the cell membrane to H_2_O_2_ is low [7, 8, 31] or the rate of H_2_O_2_ degradation is high near the cell membrane [39]. In addition, the difference in effects possibly indicates that the modulation of thiol groups takes place mainly on the intracellular side of the plasma membrane. This is further supported by the fact that there are no cysteines in the extracellular location of the Kv 2.1 channel. In Kv2.1 channels, 15 cysteines are present: four in a COOH-terminal domain, three in transmembrane core regions (S2 and S6), and the remaining eight in an NH_2_-terminal domain (Supplementary Fig. 1). Our electrophysiological data showed that the reaction of Kv2.1 with H_2_O_2_ or GSSG caused a pronounced increase in channel kinetics and left shift of steady state activation. However, all cysteines of Kv2.1 channels are located outside of S4, a central component of the voltage sensor. Given that the NH_2_ terminus has the largest number of cysteines and it has been shown to participate in channel gating [50], one or more cysteines located at the NH_2_ terminus might be involved in the effects of H_2_O_2_ on channel activation. Further studies are required to ascertain this.

Combined with the fact that endothelial cells can produce up to 500 μM H_2_O_2_ [27] and myoendothelial gap junction can be a pathway of H_2_O_2_ from the endothelium to the smooth muscle [16], our results suggest that endothelium-derived H_2_O_2_ can act as a relaxing factor in mammalian arteries. EDHFs are important factors controlling the vascular tone. Sobey [64] suggested that EDHFs play a major role in conditions of high blood pressure, arteriosclerosis, and diabetes by controlling potassium ion channels. The identity of the EDHFs differs depending on the animal species and type of arteries examined [24, 35, 41, 42, 52, 54]. The four major EDHF candidates are an electrical coupling through myoendothelial gap junctions, potassium ions (K^+^), cytochrome P450 metabolites of AA such as epoxyeicosatrienoic acid, and H_2_O_2_ [7, 31, 42, 76]. A study published in 1991, for the first time, suggested that H_2_O_2_ was an EDHF [6]: production of H_2_O_2_ by hyperpolarization of the endothelium, which consequently acts on vascular smooth muscle cells, causing relaxation of blood vessels. Subsequently, several studies have verified the hypothesis that H_2_O_2_ is an EDHF in animal and human arteries [35, 41, 42, 48, 76]. Although the contribution of EDHFs to vascular tone is not entirely clear, it is generally accepted that nitric oxide (NO) plays a dominant role in controlling the tone of large conduit blood vessels compared to EDHFs, whereas EDHF is more important in small-resistance blood vessels [14]. Consistent with this notion, we found that acetylcholine-induced endothelium-dependent vasodilation was largely inhibited by catalase in small mesenteric arteries. In contrast, acetylcholine-induced endothelium-dependent vasodilation was largely inhibited by an NO synthase blocker, but not by catalase in the aorta (Supplementary Fig. 2).

Posttranslational modifications (PTMs) are important mechanisms regulating ion channel functions. One of the classical PTMs is protein phosphorylation, and a large number of ion channels are regulated by phosphorylation through protein kinase A (PKA), PKC, and other protein kinases [19, 32, 60-62, 79]. A variety of different types of PTMs (e.g., ubiquitylation, SUMOylation, *O*-glycosylation/*O*-GlcNAcylation) exist and are discussed elsewhere [9, 13, 53, 56]. Among all these PTMs, redox-mediated PTM is an important category of PTMs that targets the thiol group of cysteine residues. Recently, redox-mediated PTMs are receiving increasing attention, as they are found in both physiological and pathological conditions, including oxidative stress. *S*-glutathionylation is a major redox-mediated thiol modulation mechanism, involving the addition of a GSH moiety to the protein. Oxidative stress and ROS facilitate *S*-glutathionylation. Over the past few years, *S*-glutathionylation has been increasingly observed in many ion channels such as voltage-gated calcium channels, the ryanodine receptor, and K_ATP_ channels, all of which contribute to critical cellular functions [1, 71, 77, 78]. Our results indicate that the Kv channel protein is significantly glutathionylated after exposure to H_2_O_2_ (Fig. 4). As delineated above, *S*-glutathionylation of the Kv channel resulted in an increase in the Kv currents in myocytes (Fig. 5). Alterations in thiol groups on proteins can alter function through structural changes in the channel protein. Since H_2_O_2_ changed the channel gating properties (Fig. 3), we propose that *S*-glutathionylation of the Kv2.1 channel protein causes a structural rearrangement of the channel that results in an increase in voltage sensitivity.

In conclusion, H_2_O_2_ relaxed rat mesenteric arteries by *S*-glutathionylation-dependent activation of Kv currents under physiological conditions. Our data suggest that *S*-glutathionylation of the Kv channel protein is, at least in part, an important and novel mechanism of 4-AP-sensitive Kv current activation by H_2_O_2_. Identifying the mechanisms underlying the vasoactive effects of H_2_O_2_ should increase our understanding of diseases where oxidative damage has been implicated such as in hypertension, atherosclerosis, and diabetes mellitus.

## Electronic supplementary material

Below is the link to the electronic supplementary material.

ESM 1 (PDF 164 kb)

## Abbreviations

4-AP: 4-Aminopyridine
AA: Arachidonic acid
BioGee: Biotinylated glutathione ethyl ester
BK_Ca_: Big-conductance Ca^2+^-activated K^+^
DTT: DL-Dithiothreitol
EDHF: Endothelium-derived hyperpolarizing factor
Em: Membrane potential
GSSG: Oxidized form of glutathione
H_2_O_2_: Hydrogen peroxide
K_ATP_: ATP-sensitive K^+^
K_Ca_: Ca^2+^-activated K^+^
Kir: Inward rectifier K^+^
Kv: Voltage-gated K^+^
MASMCs: Mesenteric arterial smooth muscle cells
NO: Nitric oxide
NT: Normal Tyrode
PSS: Physiological salt solution
ROS: Reactive oxygen species
TEA: Tetraethylammonium
