# Supplementary material for: Hydrogen peroxide induces vasorelaxation by enhancing 4-aminopyridine-sensitive Kv currents through S-glutathionylation
Source: Pflugers Arch. 2014 Apr 23;467(2):285–97. doi: 10.1007/s00424-014-1513-3 (PMC4293500; doi:10.1007/s00424-014-1513-3)
Supplement: Supplementary file 1 — (PDF 164 kb) [file 424_2014_1513_MOESM1_ESM.pdf]

Supplementary Figure 1

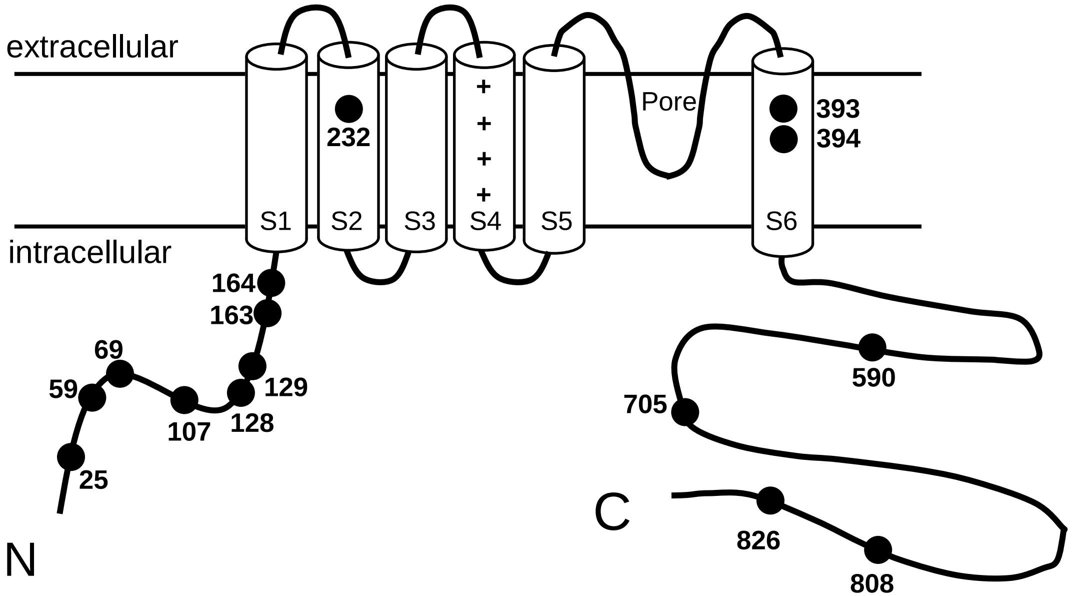

Supplementary Figure 2

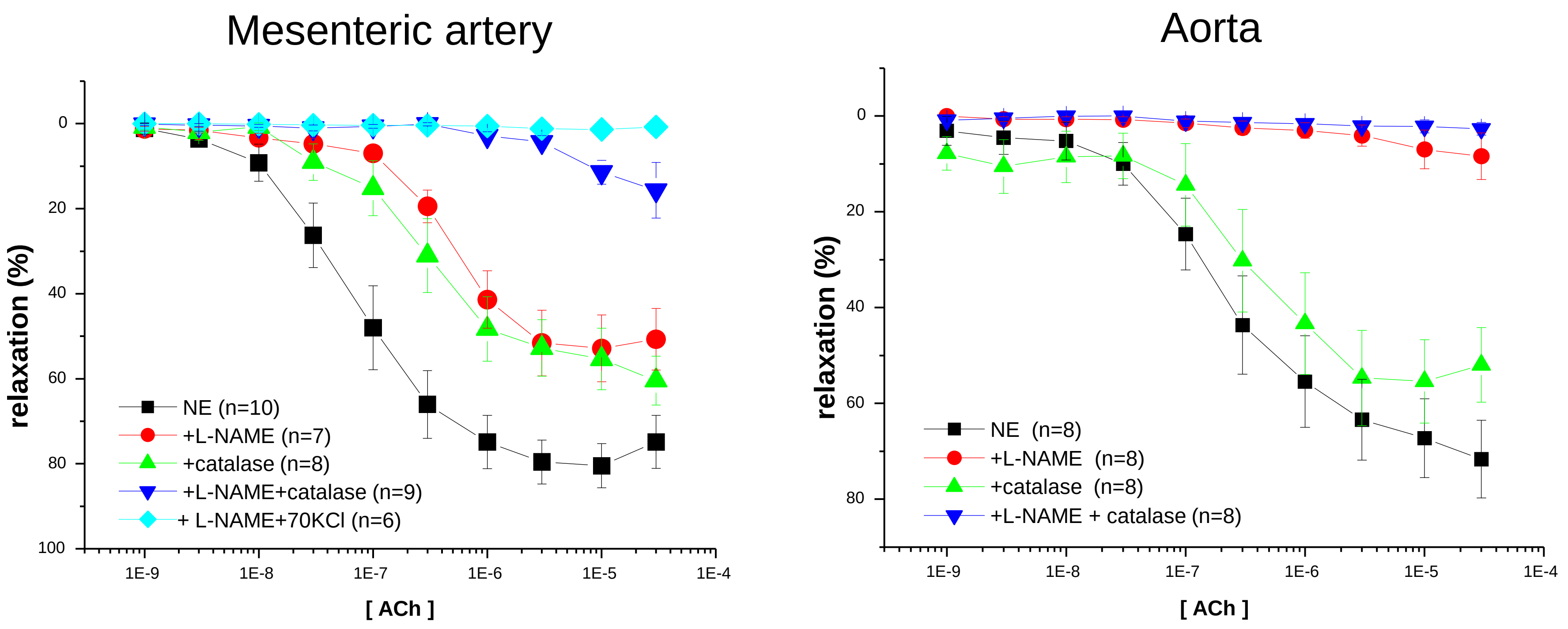

Supplementary Fig. 1. A cartoon representation shows the topology of a single rat Kv2.1 channel with all 15 cysteine residues labeled.

Supplementary Fig. 2. Roles of H_2O_2 and nitric oxide (NO) in the acetylcholine-induced, endothelium-dependent relaxation of mesenteric artery and aorta. H_2O_2 plays a primary role in small mesenteric arteries whereas NO plays a primary role in the aorta. Mesenteric arteries and aorta were precontracted with NE (10 μM). The contribution of H_2O_2 and NO to ACh-induced endothelium-dependent relaxation was determined by the inhibitory effect of catalase (500 U/mL, an enzyme which dismutates H_2O_2 to form water and oxygen) and Nω-nitro-L-arginine methyl ester (L-NAME, 100 μM; an inhibitor of nitric oxide synthase), respectively. The contribution of endothelium-dependent hyperpolarization was determined by the inhibitory effect of KCl (70 mM). Experiments were performed in the presence of indomethacin (10 μM).
